# Level of Structural Integration and Its Association with Intersession Experiences and Outcomes: A Pilot Study

**DOI:** 10.3390/ijerph19159254

**Published:** 2022-07-28

**Authors:** Thorsten-Christian Gablonski, Birgit Senft, Sylke Andreas

**Affiliations:** 1 Institute for Psychology, University of Klagenfurt, 9020 Klagenfurt am Wörthersee, Austria; sylke.andreas@aau.at; 2 Reha-Klinik für Seelische Gesundheit Klagenfurt, 9020 Klagenfurt am Wörthersee, Austria; office@statistix.at

**Keywords:** level of personality functioning, level of structural integration, intersession experiences, operationalized psychodynamic diagnosis

## Abstract

The level of structural integration (LSI), a psychodynamic/psychoanalytic concept originally developed by the Operationalized Psychodynamic Diagnosis (OPD), provides a promising empirical approach that is recognized beyond the boundaries of psychoanalysis and is highly relevant for therapy and research. The aim of our study was to investigate the intersession experiences of patients in psychotherapy with different levels of structural integration. The sample consisted of 69 inpatients who were undergoing psychotherapeutic treatment. The patients were asked to complete the German version of the Intersession Experience Questionnaire (IEQ), the short version of the OPD Structure Questionnaire (OPD-SQS) and the Brief-Symptom Inventory (BSI). LSI is associated with the situations, contents and negative emotions in the intersession experiences of patients, as well as their symptom distress over the course of therapy. Furthermore, the level of structural integration is a significant predictor of outcomes. Patients with different LSI had different intersession experiences.

## 1. Introduction

The meaning of the concept of structure has considerably changed in the history of psychoanalytic psychology and psychotherapy research. It has been taken up, adapted and reinterpreted by various psychological perspectives and different paradigms. According to Küchenhoff [1], there are three main definitions of structure in the context of psychology: (1) structure as an agglomeration of personality traits, (2) structure as described in Freud’s topographical model, and (3) structure as the level of personality functioning with time-stable basic personality functions. Structure as the level of personality functioning (LPF) or level of structural integration is the most recent definition and has become more important in recent years [2,3].

Various approaches for the assessment of level of personality functioning have been developed, not only since the publication of the fifth edition of the Diagnostic and Statistical Manual of Mental Disorders (DSM-5) [4] but also with an alternative model for the dimensional assessment of personality disorders. Zimmermann et al. [5] provided an overview of newly developed measures (self-report measurements, expert ratings, and structured interviews) according to DSM-5 section III and ICD-11. However, the Operationalized Psychodynamic Diagnostic 2 (OPD-2) [6] can be seen as a pioneer in the diagnostics of the level of personality functioning, especially in German-speaking countries. OPD-2 is an economically psychodynamic multiaxial diagnostic and classification system that was initially developed in 1996. The OPD system consists of five axes: (1) experience of illness and prerequisites for treatment, (2) interpersonal relationships, (3) conflict (4) structure, and (5) mental and psychosomatic disorders. Axis IV, which constitutes the levels of structural integration, is of especially high relevance to assess the level of personality functioning. In particular, the OPD system provides a scale called the Level of Structural Integration Axis (LSIA). The LSIA describes the level of structural integration in four dimensions and refers to the representation of self and others: (1) self-perception and object perception, (2) control of self and of relationships, (3) emotional internal communication and communication with the outside world, and (4) internal attachment and external relationships. For the assessment, the OPD system differentiates among a high level of structural integration, moderate level of structural integration, low level of structural integration and disintegrated level of structure [6]. Since the OPD system has successfully been used in research and practice for more than 20 years, numerous further developments are available. For example, Ehrenthal et al. [7] published a self-rating questionnaire on the OPD structure axis, the OPD Structure Questionnaire (OPD-SQ), and a short version of the OPD-SQ, named OPD-Structure Questionnaire Short (OPD-SQS), a few years later [8]. Jauk and Ehrenthal [9] showed that the level of structural integration as assessed in the OPD and emotional intelligence as a part of personality psychology likely assess the same latent structure so that the level of structural integration can be seen as very similar to the level of personality functioning.

Previous research has shown that the level of structural integration and level of personality functioning correlate with symptom severity and psychosocial impairments [10,11,12,13,14,15,16,17,18]. Studies have provided evidence that a low level of personality functioning is a significant predictor for successful referral to outpatient therapy [19] and higher drop-out rates [20,21]. Spitzer et al. [22] described that patients with a high level of structural integration and patients with a moderate level of structural integration had approximately the same symptom improvement with medium to high effect sizes (d = 0.70–0.83). Patients with a low level of structural integration based on the OPD had the lowest symptom improvement with a small effect size (d = 0.2). However, the results were not statistically significant [22]. A longitudinal study conducted by Bock et al. [23] showed that the levels of structural integration in adolescents according to the Operationalized Psychodynamic Diagnosis in Childhood and Adolescence (OPD-CA) [24] correlated with later symptoms and overall burden in early adulthood seven years later. The level of structural integration significantly correlated with the number of axis I diagnoses (r = 0.61, *p* < 0.0025), number of axis II diagnoses (r = 0.62, *p* < 0.0025), and global severity index (r = 0.39, *p* < 0.0125) seven years later. Furthermore, the level of structural integration is associated with more active negative emotions [25] and can be improved after successful psychotherapy [2,26]. In a study by Kraus et al. [2], patients had an OPD-SQ global score of 2.25 (0.53) at the beginning of therapy. After successful therapy, they improved with an OPD-SQ global score of 1.95 (0.62) at the end of therapy [2]. Currently, there are no cut-off-scores for the OPD-SQ, but Ehrenthal et al. [7] provided information about the mean scores of different populations in the development of the questionnaire. In their investigation, at the beginning of treatment, participants from the nonclinical sample had an OPD-SQ mean-score of 1.25 (0.48); outpatients had a mean-score of 1.68 (0.56); and inpatients had an OPD-SQ mean-score of 2.04 (0.57).

Horowitz [27] described that psychotherapeutic treatment of patients with a low level of personality functioning can be very challenging, since they are more vulnerable. Therefore, it is necessary to use alternative therapeutic techniques to avoid the negative effects of patients feeling criticized by confrontative interpretations by the therapist [27].

Negative feelings related to psychotherapy can promote negative treatment progress, especially when negative feelings occur not only during a session but also between therapy sessions. Studies have shown that the thoughts and feelings related to psychotherapy between therapy sessions have a significant impact on the treatment progress and outcomes. These processes are called intersession experiences (ISEs) [28,29]. Intersession experiences or intersession processes are representations of the therapy or the therapist and describe all forms of intentional thoughts, feelings, memories, and fantasies about therapy and the therapist between two therapy sessions [28,29]. Intersession experiences can be described by various factors. For example, how intensively did someone think about therapy between the sessions? Intersession experiences can also be described by the situations and contents of intersession experiences as well as different emotions. For example, a patient suffering from a mild anxiety disorder travels to work by train every day. These journeys are unpleasant for him/her. To calm down, he/she thinks about what he/she discussed with the therapist during the last session (recreating therapeutic dialogue) during the 10 m ride. This action gives him/her a feeling of security and encourages him (positive emotions).

According to Orlinsky and Geller [29], the theoretical model of intersession experiences is based on various psychoanalytic and developmental psychological theories that include the concepts of internalization and representation, since intersession experiences can be seen as representations of therapy and the therapist. Therefore, their conceptualization represents selective integration of these two theoretical traditions. The concept of intersession experiences can be acknowledged in the Generic Model of Psychotherapy [30,31], a comprehensive transtheoretical model that integrates different results of psychotherapy research. The Generic Model of Psychotherapy differentiates between input, process, and output variables on three different levels. The first level includes input variables, such as patient characteristics, social factors and the involved health system. On the second level, there are process variables that directly influence outcome variables on the third level. For example, process variables are the therapeutic relationship or therapeutic techniques, and change processes over time. These temporal characteristics can be expressed as micro-outcomes within a therapy session or as a macro-outcome over the entire course of a therapy. In the Generic Model of Psychotherapy, intersession experiences are located between post-session results and the short-term results of therapy [32].

Since its conceptualization, there have been only few research projects on intersession experiences [33]. However, previous results have clearly shown that there is a high correlation between ISEs and therapeutic alliance [34,35,36,37] and between ISEs and outcomes [37,38,39,40,41]. Hartmann et al. [41] showed in a study with bulimic patients who intersession processes are a better predictor of outcomes, including the therapeutic relationship itself.

Disorder-specific studies related to intersession experiences have shown that the type of mental illness has an impact on the intersession experiences of patients. For example, a study of Bender et al. [42] in patients with specific personality disorders (schizotypal, borderline, avoidant and obsessive-compulsive) investigated the representations of these patients compared to those of a control group of patients with major depression. The results showed that patients with schizotypal personality disorder most intensively deal with therapy outside the therapy setting, miss the therapist more than other patients and want to become friends with the therapist more urgently [42]. However, patients with borderline personality disorder have the greatest difficulties with the representation of a harmless therapist [42]. Zeeck et al. [43] examined differences between neurotic patients and patients with borderline personality disorder and showed that patients with borderline personality disorder have more intersession experiences with more negative emotions over the entire course of treatment. In addition, the researchers showed that patients with borderline personality disorder, especially those who are in the first and middle stages of the course of treatment, have significantly more frequent relationship fantasies involving the therapist than neurotic patients [43]. Based on these results, we assume that patients with a low level of personality functioning have the greatest difficulties with intersession experiences. It might be that the limited structural functions of these patients, including overwhelming emotions, can cause worse and more difficult intersession experiences than in patients with good structural integration.

Although studies on the intersession experiences of patients with different personality disorders are available, no research has addressed the relationship between the level of structural integration and the intersession experiences of patients. However, investigating the intersession experiences of patients with different levels of structural integration might help us more clearly understand why patients with different levels of personality functioning and different experiences in therapy have that need to be targeted.

Therefore, the aim of this pilot study was to examine whether different levels of structural integration are associated with different intersession experiences over the course of therapy and to examine if there are differences regarding outcomes. The results could be the basis for further studies on the level of personality functioning and intersession experiences from a psychodynamic perspective. Thus, this pilot study investigated the following three hypotheses:

**Hypothesis** **1.** *The patient’s level of structural integration is a significant predictor of intersession experiences during a psychotherapeutic treatment. Based on the study results presented above, it can be assumed that patients with a lower level of structural integration have more negative feelings and more intensive intersession experiences. These negative feelings could have an impact on the course of treatment and outcomes. If patients with a low level of structural integration have more negative feelings between sessions, it is important to address this problem and consequently improve the outcomes.*

**Hypothesis** **2.** *The patient’s level of structural integration is a significant predictor of symptom distress. As mentioned above, previous studies have demonstrated significant correlations between a patient’s level of structural integration and symptom distress. We assume we will replicate these results.*

**Hypothesis** **3.** *The patient’s level of structural integration is a significant predictor of outcomes. Based on previous study results, we assume we will find a positive correlation between the level of structural integration and outcomes. The better the patient’s level of structural integration, the better the outcome.*

## 2. Methods

### 2.1. Design

We conducted a study with two measurement points at the beginning (T1) and end (T2) of therapy to have pre- and post-assessments for outcomes with a consecutive sample. In collaboration with a rehabilitation hospital in Carinthia (Klagenfurt, Austria) over a period of 15 weeks, all inpatients were asked to complete questionnaires. The psychosomatic rehabilitation clinic is mainly for patients with depressive, anxiety and psychosomatic disorders and offers a wide range of different treatments. The length of hospital stay is six weeks and includes individual (once per week) and group psychotherapy (twice per week), medical specialist care, psychoeducation, occupational therapy, and physical education, among other things. The clinic admits patients cyclically, which means that each patient’s cycle begins and ends at the same time. The described setting was particularly suitable to answer our research questions, as it was possible to recruit a sufficiently large sample with patients who could be compared with each other. In addition, the inpatient setting with frequent therapy sessions could be compared to a traditional psychoanalytic treatment with a high frequency.

During the aforementioned observation period, the patients completed the short version of the OPD Structure Questionnaire (OPD-SQS) [8] and Brief Symptom Inventory (BSI) [44] within the first week after admission. Immediately prior to the second individual therapy session (usually in the second week), (T1), the patients received the German version of the Intersession Experience Questionnaire (IEQ) [45]. In the sixth and final week of hospitalization, the patients completed the IEQ and BSI immediately before the final individual therapy session (T2). Therapists were mainly trained in cognitive behavioral therapy. Before the study, the patients were informed about the study verbally and in writing. In addition, signed informed consent forms were obtained from the patients. The study was conducted in accordance with the principles of the Declaration of Helsinki.

### 2.2. Instruments

Intersession Experience Questionnaire (IEQ): The Intersession Experience Questionnaire was used to measure the intersession experiences of patients. The German version of the Intersession Experience Questionnaire [45] consists of 48 items (and 4 additional open-ended items) on the following 5 subscales:

(A) Intensity of intersession experiences
(B) Context of intersession experiences
(C) Content of intersession experiences
(D) Emotional quality of intersession experiences (D1: positive emotions; D2: negative emotions)
(E) Significant others, sharing intersession experiences

Immediately before a therapy session, patients rated their intersession experiences on a 5 point Likert scale. The questions relate to the period between the previous and the current session. It took patients approximately five to ten minutes to complete the questionnaire. Hartmann et al. [45] investigated the psychometric properties of the German version of the IEQ. We examined the internal consistency in our sample. In our sample, the internal consistency of most factors on the IEQ was satisfactory or good for T1 and T2 (A: α = 0.754/0.779; B: α = 0.710/0.882; C: α = 0.812/0.834; D1: α = 0.848/0.876; D2: α = 0.769/0.856; and E: α = 0.634/0.734). The last subscale was ignored in this study due to poor internal consistency.

OPD Structure Questionnaire Short (OPD-SQS): Based on the OPD-SQ [7], Ehrenthal et al. [8] developed a short version of the questionnaire (OPD-SQS) [8]. The OPD-SQS consists of only 12 items. Higher scores on the OPD-SQS indicate lower levels of structural integration. The extraction and model verification resulted in a three-factor model. The short version has the following subscales [8]:

(A) Self-awareness
(B) Interpersonal behavior
(C) Relationships

The psychometric properties of the short version of the questionnaire were satisfactory [8]. The 12 items had good internal consistency (Cronbach’s α = 0.88). Additionally, inpatients had a higher score than outpatients (Cohen d = 1.35) and patients without treatment (Cohen d = 0.62), which indicates criterion validity [8]. In our sample, the internal consistency of OPD-SQS (self-awareness: α = 0.736; interpersonal behavior α = 0.650; and relationships: 0.835) was satisfactory.

Brief Symptom Inventory (BSI): The Brief Symptom Inventory (BSI) [44] is a common, reliable and valid self-report measure designed to assess symptom patterns and severity of patients in the prior seven days. It is a short version of the Symptom Checklist (SCL) and consists of 53 items on nine scales (somatization, obsessive-compulsive, interpersonal, depression, anxiety, hostility, phobia, paranoia, and psychoticism). Global psychological distress is indicated by the Global Severity Index (GSI). All items are rated on a 5 point Likert scale. The BSI has good internal consistency ranging from α = 0.70 to α = 0.96. The highest internal consistency was found for the GSI (α = 0.96) [44]. In our sample, the internal consistency for the GSI was α = 0.942/0.936.

### 2.3. Sample

During the 15 week survey period, consecutive inpatients who agreed to participate in the study and provided informed consent were included in the sample. During this period, three patient cycles were considered with an overall sample size of 115.

Due to various reasons (discontinuation, patient noncompliance, and organizational failures), only 60% (*n* = 69) of the patients completed both the OPD-SQS and at least the IEQ at the beginning and/or the end of therapy. This participation was the minimum requirement for inclusion. Patients who did not complete the OPD-SQ and the ISQ at least once were excluded (*n* = 46). There were no differences in age (F (1, 113) = 2.215, *p* = 0.140, η^2^ = 0.019) or gender (F (1, 113) = 0.631, *p* = 0.429, η^2^ = 0.006) between patients who were included and those who dropped out. Of the 46 patients who were excluded, 36 did not complete the OPD-SQS at the beginning of therapy due to organizational failures. The final sample consisted of 69 patients from 18 different therapists. The therapist with the highest number of patients treated 9 of the 69 patients.

On average, the patients were 44 years old. Approximately 62% (*n* = 43) of the patients were female, whereas 38% (*n* = 26) were male. The most common diagnoses (rated by experts from the hospital based on ICD-10 [46] were major depressive disorder (recurrent, moderate), post-traumatic stress disorder and major depressive disorder (recurrent, mild) (Table 1).

### 2.4. Statistical Methods

Due to repeated measures, a longitudinal multilevel modeling approach to the data was deemed appropriate for data analyses. Our data structure encompassed repeated measurements (Level 1) nested within the individual (Level 2). Separate multilevel models were run to predict intersession experiences and symptom distress with the patient’s level of structural integration (research question 1 + 2). The applied multilevel model is expressed in the following general equation:

$$IEQ_{ti}=\beta_{0}+\beta_{1}\left(time\right)+\beta_{2}\left(OPD-SQS\right)+\left[\mu_{oi}+e_{ti}\right].$$

$IEQ_{ti}$ represents a patient’s (*i*) intersession score at time *t*. $\beta_{0}$ is the average intercept, which can vary between patients ($\mu_{oi}$). $\beta_{1}$ is the time predictor variable, and $\beta_{2}$ is the time-invariant variable. Finally, $e_{ti}$ reflects the time-specific error term. In our analyses, we only used the random intercept, meaning that the average intersession score over time was varied by person and a random slope that allowed individual variability among the patients. We used the Akaike Information Criterion (AIC) [47] for model comparisons. The statistical analysis was performed with R version 3.5.2. [48]. For the multilevel modeling approach, we used the R package lme4 [49].

For research question 3, we used the Reliable Change Index (RCI) [50] to operationalize outcomes. The RCI shows whether the magnitude of change is statistically reliable. In our study, the RCI of all patients was assessed for the BSI as a central outcome parameter using the following formula according to Jacobson and Truax [50]:

$$RCI=\frac{\bar{X_{1}}-\bar{X_{2}}}{SE}$$

$\bar{X_{1}}$ represents the pretest score at admission, and $\bar{X_{2}}$ represents the posttest score at discharge. SE was calculated as follows:

$$SE=sd\times \sqrt{1-r_{tt}}$$

where sd represents the standard deviation of the control group or the normal population and $r_{tt}$ represents the test-retest reliability. For our analyses, we used the published scores in the BSI manual [44], in which the standard deviation of the normal population is described as sd = 0.72 and the test-retest reliability is described as $r_{tt}$ = 0.90 [44]. This strategy resulted in the following formula for our analyses:

$$RCI=\frac{\bar{X_{1}}-\bar{X_{2}}}{0.228}$$

The results were classified in three subgroups according to Jacobson and Truax [50], who defined an RCI greater than 1.96 as a positive reliable change, an RCI less than −1.96 as a negative reliable change and scores between these values as an absence of change. Subsequently, an ordinal logistic regression analysis was performed to assess whether the OPD level of structural integration was a significant predictor of outcomes. The statistical analysis was performed with SPSS version 25 [51].

## 3. Results

Descriptive statistics for the IEQ, OPD-SQS and BSI are presented in Table 2. The OPD-SQS global mean score in our sample was 2.04 (0.76) and, thus, as high as the mean score of the inpatient sample in a study by Ehrenthal et al. [7]. Except for negative emotions, patients showed lower intersession activity at the end of therapy (t2). At discharge, patients reported a higher score on the scale negative emotions regarding their intersession experiences. However, they showed a lower symptom burden on all scales of the BSI as well as on the total burden (GSI).

Table 3 shows the single correlations between the level of structural integration (OPD-SQS overall score) and the single IEQ scales.

To investigate our hypotheses, we first examined the unconditional means model or base model/null model. The different null models show how much the intercept differs from baseline. As Table 4 depicts, patients reported a medium intersession intensity since the prior session (M = 2.59, SD = 0.08) and more positive emotions regarding therapy and/or the therapist (M = 2.41, SD = 0.08) than negative emotions (M = 1.10, SD = 0.09). Next, we added the time variable as a predictor in our model. As Table 4 indicates, there was a significant change in the intensity and symptom distress of the patients between the two measurement points (i.e., at the end of treatment, patients reported significantly lower intensity of intersession experiences (*β* = −0.28, *SE* = 0.09, *t* = −2.94, *p* = < 0.01) and symptom distress (*β* = −0.26, *SE* = 0.07, *t* = −3.75, *p* = < 0.01)). Last, we added the patient’s level of structural integration to the model. Except for the IEQ scale positive emotions, every model improved after adding the time predictor and the patient’s level of structural integration. Therefore, the patient’s level of structural integration during the course of therapy (time variable) was a highly significant predictor of the situation of intersession experiences (*β* = 0.26, *SE* = 0.11, *t* = 2.24, *p* = < 0.05), contents of intersession experiences (*β* = 0.19, *SE* = 0.01, *t* = 2.10, *p* = < 0.05), negative emotions in the intersession period (*β* = 0.43, *SE* = 0.11, *t* = 3.98, *p* = < 0.01) and symptom distress measured by the global severity index (*β* = 0.53, *SE* = 0.09, *t* = 5.82, *p* = < 0.01).

For hypothesis 3, we first calculated the reliable change index (RCI) for all patients and classified them in different outcome groups based on their RCI. Based on the RCI, 27.5% of the patients were classified as recovered, whereas 58.0% were classified as unchanged. Only 4 (5.8%) patients were classified as deteriorated. The ordinal logistic regression analysis showed that the OPD level of structural integration as a predictor of outcomes leads to a significant improvement in the fit of the final model over the null model (χ^2^ (1) = 6.811, *p* = 0.009). In line with this finding, the patient’s level of structural integration was a significant predictor of outcomes (*β* = 0.971, SE = 0.405, *p* = 0.016).

## 4. Discussion

Psychoanalytic psychotherapy has been criticized for years due to the lack of empirical evidence. Since psychoanalysis regards the individuality of a person as a central component, there is only a limited willingness for empirical research in terms of DSM and ICD labels. Due to dissatisfaction with existing descriptive classification systems, the OPD was developed and published. The OPD level of structural integration axes provides an empirical approach for the classification of individual patient characteristics without neglecting the uniqueness of the person. This form of diagnostics has also gained acceptance beyond psychoanalysis. In recent years, it has received increasing attention in research and practice, not least because of recent developments in PD diagnostics. Therefore, research will have to deal with the question about characteristics of patients with different levels of personality functioning and their relevance for the psychotherapy process and outcomes in the future. The present pilot study investigated the relationship between the patient’s level of structural integration and intersession experiences and outcomes.

The first hypothesis in the study addressed the relationship between the level of structural integration (LSI) and intersession experiences. The mean scores of the OPD-SQS in our sample corresponded to the mean scores in other studies with clinical samples [8]. We found that the patient’s level of structural integration was a significant predictor for the situation, contents and negative emotions of intersession experiences during inpatient psychotherapy (accounted for time). The results show that patients with a poorer level of structural integration have more intersession experiences. These results correspond with previous study results. Zeeck et al. [43] investigated the differences in the intersession experiences of neurotic and borderline patients. They observed that in the beginning and middle stages of therapy, patients with borderline personality disorder reported higher scores on the IEQ. Especially in relation to negative emotions, patients with borderline personality disorder had significantly more intersession experiences over the entire course of therapy [43]. Comparisons to this study are legitimate. Regarding clinical experience, patients with borderline personality disorder usually have a lower level of personality functioning than neurotic patients [52].

Regarding the OPD-2 [6] definitions of structural levels, patients with a high level of structural integration have a repertoire of psychological tools for a differentiated perception of mental experience. However, due to their incoherent self and the overflowing emotionality that they experience, patients with a low level of structural integration are unable to adequately perceive mental experiences [6]. Therefore, patients with low levels of structural integration are unable to have a good and stable therapeutic relationship from the beginning of therapy, because they test the therapeutic relationship and the limits and trust of their therapists [53]. This problem is also closely linked to deficient object constancy in patients with borderline personality disorder. Object constancy is the ability to maintain an emotional bond with other individuals even when they are not physically present, an ability that is limited in patients with borderline personality disorder [54,55,56]. These patients may need to complete substantial mental homework and think about therapy or the therapist, since the objects of therapy and therapist are not internally represented. Deficient object constancy is often compensated by a strong attachment to transitional objects [55,56]. This phenomenon could be a potential explanation for the results in this study, which showed that patients with a low level of personality functioning reported more frequent intersession experiences. The structural capabilities described by the OPD Task Force [6] can be cited as a potential explanation of the patterns described in this study. Accordingly, it can be assumed that patients with a high level of structural integration perform more efficient intersession work with their available regulatory functions and their differentiated perception of their mental experiences. Therefore, the ultimate effects of therapy, reflections, internalization, thoughts and feelings could be more rapidly and adequately processed, which means a lower frequency of intersession experiences than that in patients with a low level of structural integration.

The results associated with the second hypothesis show that the level of structural integration is a highly significant predictor of symptom distress. This result corresponds to previous results [57] and shows that patients with a low level of structural integration have higher symptom distress. Overall, it can be assumed that the level of structural integration is closely related to symptom distress. This result confirms the results of previous studies [10,11,12,13,14,15,16,17,18]. In general, it is unclear to what extent these two concepts may at least partially measure the same latent construct.

The investigation of our third hypothesis showed that the level of structural integration is a significant predictor of outcomes operationalized as a reliable change based on the recommendation of Jacobson and Truax [50]. In our sample, patients with a lower level of structural integration were more likely to reach a reliable change after the six weeks of treatment than patients with a higher level of structural integration. The six week clinical stay probably allowed patients with higher initial symptom severity to achieve more improvement compared to patients who had lower symptom severity at the beginning of therapy.

## 5. Limitations

This pilot study has several limitations. In particular, the sample size was small for the large number of investigated variables. Furthermore, the patients in this study had both weekly individual psychotherapy and frequent group psychotherapy. The intersession questionnaires were related to the individual setting and not to the potentially more frequently occurring group therapy sessions. Therefore, intersession experiences in association with group psychotherapy sessions could yield other results. Andreas et al. [37] investigated patients’ intersession experiences related to individual therapy and group therapy. They found significant differences in intersession experiences for individual therapy and group therapy and a significant relationship between intersession experiences and outcomes and the therapeutic alliance. It was not possible to control the specific therapeutic interventions in this study. Consequently, different therapists treated patients in an individual setting. In addition, patients received different treatments, depending on the training of their therapist. According to the results of Owen et al. [34], therapeutic orientation could have an impact on intersession experiences and outcomes. For example, psychoanalysis could induce different forms of intersession experiences than behavioral therapy. Moreover, we used only self-reports in this study. Expert ratings are more suitable to capture symptomatic changes and outcomes. Therefore, future studies should also include expert ratings. Furthermore, it is necessary to mention the weaknesses of the questionnaires used in this study. As a recent study has shown, OPD-SQS assesses not only the level of structural integration, but also other aspects of psychopathology (e.g., depression) [58]. This limitation was critical for this study. Obbarius et al. [58] described that the OPD-SQS factor self-efficacy is affected by current depression, stress and anxiety. Consequently, the level of personality functioning score in our study was affected by the current day’s state, which is different from the normally stable patient’s level of personality functioning. Therefore, it is necessary to repeat our study with better instruments, such as an OPD interview. Last, we were unable to examine if the patients who dropped out significantly differed from our sample regarding the level of structural integration. These points must be considered when interpreting the data.

## 6. Conclusions, Clinical Implications and Implications for Further Research

Our study provides the first indication for the association between the level of structural integration and aspects of intersession experiences. We can conclude that patients with different levels of structural integration show different mental representations of therapy and their therapists. We do not know why patients with a lower level of structural integration reported more intersession experiences. It might be that patients with a lower level of structural integration need more support, including between therapy sessions. It could be of high importance to provide them the chance to talk about previous sessions and to support them with their mental representations. The highest correlation was found between the level of structural integration and negative emotions in intersession experiences. In clinical practice, it is important to work on these negative emotions. Therapists should support their patients to handle and overcome their negative emotions in the context of their intersession experiences. As some studies have shown, intersession experiences are associated with treatment outcomes. Therefore, the promotion of intersession experiences, especially for patients with higher levels of structural integration, could benefit the treatment course and outcomes. Additional studies with larger samples could try to replicate the results and obtain more statistically significant results. Moreover, a third follow-up time point could be added. In addition, it would be interesting to collect data on variables that are directly related to therapy, such as the motivation to seek treatment. This strategy could clarify the possible connection between treatment motivation and intersession experiences. Further studies should analyze intersession experiences with time series analysis (measurement of intersession experiences before every therapy session) or ambulatory assessments in which intersession experiences are associated with direct events in therapy. It would also be extremely useful to assess the actual relatively stable level of structural integration of a patient at the end of therapy to control for whether intersession experiences are caused by potential changes in the structure. It is widely unclear what kind of in-session processes promote good or helpful intersession experiences. Transference could be a relevant concept to understand the mechanism of intersession experiences. In the current study, we investigated the association between in-session experiences operationalized by control mastery theory [59] (CMT), mentalization and intersession experiences. We assume that transference processes and situations, in which the patients test their pathogenic beliefs in the relationship with their therapists, might have an impact on the reflection functioning of patients and their intersession experiences. The mentalizing ability of patients, as a foundation to understand their own mental processes, could be an important concept to understand the effect mechanism of the intersession experiences of patients with mental disorders and to promote understanding of intersession experiences and their association with the level of structural integration.

Another important approach in the future could include the use of smartphone applications to collect data on intersession experiences in an efficient way. Gablonski et al. [60] published the first smartphone application framework to assess intersession experiences. To improve new measurements, additional basic investigations are required to understand the intersession experiences of patients with different levels of structural integration in a better and more differentiated way.

## Figures and Tables

**Table 1:** Sample characteristics.

		*n* = 69
		*n* (%)
Age	mean	43.6
	standard deviation	9.3
	minimum	18
	maximum	61
Gender	male	26 (37.7)
	female	43 (62.3)
Primary Diagnosis (ICD-10)	F33.1 Major depressive disorder, recurrent, moderate	19 (27.5)
	F43.1 Post-traumatic stress disorder	13 (18.8)
	F33.0 Major depressive disorder, recurrent, mild	8 (11.6)
	F32.1 Major depressive disorder, single episode, moderate	7 (10.1)
	F43.22 Adjustment disorder with anxiety	4 (5.8)
	other	18 (26.1)
Marital status	single	17 (24.6)
	divorced, separated	15 (21.7)
	married	22 (31.9)
	partnership	10 (14.5)
	other	5 (1.4)
Education	main school	33 (47.8)
	secondary school	8 (11.6)
	high school	13 (18.8)
	College	10 (14.5)
	other	5 (7.2)

**Table 2:** Descriptive data on OPD Structure Questionnaire Short (OPD-SQS), Intersession Experience Questionnaire (IEQ), and Brief Symptom Inventory (BSI).

	T1			T2		
	*n*	Mean	SD	*n*	Mean	SD
OPD Self-awareness	69	1.88	0.94	-	-	-
OPD Interpersonal Behavior	69	2.00	0.88	-	-	-
OPD Relationships	69	2.23	1.11	-	-	-
OPD Overall score	69	2.04	0.76	-	-	-
IEQ A: Intensity	55	2.79	0.72	64	2.47	0.79
IEQ B: Situation	55	1.57	0.74	64	1.35	0.89
IEQ C: Content	55	1.27	0.61	64	1.21	0.63
IEQ D1: Positive Emotions	54	2.45	0.75	64	2.42	0.75
IEQ D2: Negative Emotions	53	1.00	0.74	64	1.13	0.88
BSI Somatization	68	1.15	0.76	64	1.02	0.76
BSI Obsessive-compulsive	68	1.86	0.98	64	1.49	0.97
BSI Interpersonal	68	1.72	0.96	64	1.33	0.94
BSI Depression	68	1.68	0.97	64	1.28	0.80
BSI Anxiety	68	1.70	0.98	64	1.48	0.97
BSI Hostility	68	1.07	0.72	64	0.92	0.70
BSI Phobia	68	1.36	1.12	64	1.03	1.05
BSI Paranoia	68	1.39	1.00	64	1.12	0.84
BSI Psychoticism	68	1.22	0.89	64	1.02	0.88
BSI Global Severity Index	68	1.48	0.77	64	1.22	0.71

Note. T1 = admission; T2 = discharge; Mean = mean score, SD = standard deviation.

**Table 3:** Correlation matrix between the OPD-Structure Questionnaire Short (OPD-SQS) and Intersession Experience Questionnaire (IEQ) for T1 and T2.

	OPD-SQS	Intersession Experience Questionnaire t1					Intersession Experience Questionnaire t2				
	Overall	IEQ_A	IEQ_B	IEQ_C	IEQ_D1	IEQ_D2	IEQ_A	IEQ_B	IEQ_C	IEQ_D1	IEQ_D2
**OPD_overall**	-	0.19	0.21	0.20	0.15	0.44 *	0.27	0.33 **	0.31 *	−0.17	0.36 **
**IEQ_A t1**	-	-	0.51 **	0.50 **	0.45 **	−0.01	0.56 **	0.54 **	0.59 **	0.25	0.07
**IEQ_B t1**	-	-	-	0.49 **	0.62 **	0.31 *	0.43 **	0.74 **	0.70 **	0.25	0.26
**IEQ_C t1**	-	-	-	-	0.33 *	0.26	0.50 **	0.48 **	0.78 **	0.27	0.21
**IEQ_D1 t1**	-	-	-	-	-	0.15	0.31 *	0.49 **	0.46 **	0.54 **	0.10
**IEQ_D2 t1**	-	-	-	-	-	-	0.24	0.44 **	0.36 *	−0.19	0.72 **

Note. * *p* < 0.05; ** *p* < 0.01; OPD_overall: OPD-SQS overall score; IEQ_A: intensity of intersession experiences; IEQ_B: context of intersession experiences; IEQ_C: content of intersession experiences; IEQ_D1: positive emotions; IEQ_D2: negative emotions.

**Table 4:** Results of the multilevel modeling for the prediction of intersession experiences and symptom distress.

		Model 1 (Base Model)				Model 2: Predizctor Time				Model 3: Predictor Time + OPD-SQS			
		*β* (SE)	t	AIC	ICC	*β* (SE)	t	AIC	ICC	*β* (SE)	t	AIC	ICC
IEQ	Intensity	2.59 *** (0.09)	30.55	263.62	0.55	−0.28 ** (0.09)	−2.94	261.16	0.88	0.21 (0.11)	1.94	259.44	0.87
	Situation	1.43 *** (0.09)	15.36	261.74	0.70	−0.15 (0.09)	−1.59	260.99	0.90	0.26 * (0.11)	2.24	258.22	0.90
	Content	1.24 *** (0.07)	17.35	183.84	0.77	−0.07 (0.06)	−1.13	188.56	0.91	0.19 * (0.01)	2.10	186.26	0.90
	Pos. emotions	2.41 *** (0.08)	28.67	253.75	0.59	−0.01 (0.10)	−0.14	259.68	0.87	−0.06 (0.11)	−0.54	261.41	0.87
	Neg. emotions	1.10 *** (0.10)	11.60	256.49	0.73	0.11 (0.08)	1.34	257.04	0.91	0.43 *** (0.11)	3.98	244.51	0.90
BSI	Global Severity Index (GSI)	1.36 *** (0.08)	16.39	267.78	0.66	−0.26 *** (0.07)	−3.75	259.95	0.88	0.53 *** (0.09)	5.82	234.99	0.86

Note. OPD-SQS = OPD Structure Questionnaire Short; IEQ = Intersession Experience Questionnaire; BSI = Brief Symptom Inventory * *p* < 0.05, ** *p* < 0.01, *** *p* < 0.001, *β* = standardized regression coefficient; SE = standard error; AIC = Akaike Information Criterion; ICC = intraclass correlation coefficient.

## Data Availability

The data presented in this study are available on request from the corresponding author. The data are not publicly available due to privacy restrictions.
